# Suggestive genome-wide associations with inflammatory biomarkers in an admixed population, including a missense variant in the *OR6K6* olfactory receptor gene associated with MCP-1

**DOI:** 10.3389/fimmu.2026.1876244

**Published:** 2026-08-25

**Authors:** Jean Michel R. S. Leite, Gabriela F. L. Pascoal, Graziela B. S. Duarte, Andressa Cerqueira, Regina M. Fisberg, Flavia M. Sarti, Marcelo M. Rogero

**Affiliations:** 1 Department of Nutrition, School of Public Health, University of São Paulo, São Paulo, Brazil; 2 Department of Statistics, Federal University of São Carlos, São Paulo, Brazil; 3 School of Arts, Sciences and Humanities, University of São Paulo, São Paulo, Brazil

**Keywords:** cytokines, genetic admixture, GWAS, inflammation, missense variant

## Abstract

**Background:**

Chronic low-grade inflammation drives cardiometabolic diseases and has a strong genetic basis. Most genome-wide association studies (GWAS) have focused on European populations, limiting knowledge of the genetic influences on inflammation in admixed populations such as those in Brazil.

**Methods:**

This study is part of the cross-sectional ISA Capital Health Survey. It uses data from the 2015 ISA Nutrition cohort, which measured biochemical, genetic, anthropometric, and lifestyle factors in a probabilistic sample of São Paulo residents. Genomic DNA was extracted from 841 individuals. Genotyping was performed using the Axiom 2.0 Precision Medicine Research Array. After quality control and missing data exclusion, 244,338 SNPs from 638 individuals remained for GWAS-based association analysis with eight inflammatory biomarkers. Models were adjusted for sex, age, age^2^, overweight, and the first two principal components of ancestry.

**Results:**

Most participants were male (53%) and not overweight (55%). The median age was 49, and 38% were older adults. In the genome-wide analysis of TNF-α, IL-10, IL-1β, monocyte chemoattractant protein-1 (MCP-1), and adiponectin, 12 SNPs were significantly associated, most of which were intronic. Notably, one signal mapped to the missense variant rs16841009 in the olfactory receptor gene OR6K6. This variant was associated with MCP-1, suggesting a possible involvement in inflammatory responses.

**Conclusions:**

We identified new SNPs linked to inflammatory biomarkers in a highly admixed Brazilian population, including a missense variant in an olfactory receptor gene linked to MCP-1. This association may be biologically important for inflammation and could affect the risk of cardiometabolic diseases.

## 1 Introduction

Cardiometabolic diseases (CMD), especially obesity, atherosclerosis, and type 2 diabetes (T2D), represent significant public health concerns, contributing to high morbidity and mortality, and an extensive socioeconomic burden globally (1–4). These conditions, as well as their cardiometabolic risk factors, are influenced by socioeconomic determinants, lifestyle (including diet and physical activity), and common genetic variation (5, 6).

Among the major risk factors underlying those conditions, chronic low-grade systemic inflammation plays a major role in the pathophysiology and progression of these phenotypes, and is orchestrated by a complex set of inflammatory biomarkers such as tumor necrosis factor (TNF)-α, IL (interleukin)-1β, IL-6, IL-10, monocyte chemoattractant protein (MCP)-1, adiponectin, among several others (7, 8). These cytokines and other inflammatory molecules have a considerable underlying genetic component, as shown by heritability estimates, defined as the proportion of phenotypic variance attributable to genetic effects transmitted across generations, that can reach up to 39% (9–11). Furthermore, candidate-gene studies have consistently shown associations of single nucleotide polymorphisms (SNPs) in inflammatory biomarkers, including IL-1β, IL-10, IL-6, C-reactive protein (CRP), and adiponectin, with metabolic risk and systemic inflammation (12–14).

Building upon those investigations, genome-wide association studies (GWAS) have emerged as a powerful tool for uncovering the genetic architecture of complex traits. These studies have enabled the discovery of a plethora of SNPs associated with a wide range of cardiometabolic phenotypes such as body mass index (BMI), lipid profile, coronary artery disease and several inflammatory biomarkers including CRP and multiple cytokines (15–19). However, most GWAS research has been conducted predominantly in European populations (e.g. as of 2025, 87.77% of GWAS participants are of European ancestry), leaving a gap in our understanding of how genetic variants influence inflammation in ethnically admixed populations, such as those in Brazil (20–22).

The Brazilian population is among the most genetically admixed in the world, due to its history of colonization and migration, and is mainly composed of European, African and Native-American ancestries (23–25). Recent studies in this population have unraveled the genetic components of cardiometabolic traits, including lipid profiles and atherogenic traits (16, 26). Nonetheless, to our knowledge, no study has investigated the genetic underpinnings of inflammation in this population under a genome-wide framework. Hence, this study aimed to bridge the aforementioned literature gaps by performing GWAS of inflammatory traits in a highly admixed population from São Paulo, Brazil, as part of the ISA Nutrition 2015 study. By examining these traits, we aim to identify the genetic factors underlying inflammatory modulation in a population historically underrepresented in genomic research.

## 2 Materials and methods

### 2.1 Study design and population

This investigation is part of the cross-sectional observational study “ISA Capital Health Survey”, which comprised probability-based samples of individuals aged 12 years and older living in the city of São Paulo (Brazil). Particularly, this study used data from the 2015 cohort, namely the “2015 Health Survey of São Paulo with focus on Nutrition - 2015 ISA-Nutrition”, which collected information on a comprehensive set of biochemical and genetic markers as well as socio demographic, anthropometric, lifestyle, and health care utilization variables.

The 2015 ISA-Nutrition used multistage, stratified sampling (urban census tracts and households) to create a representative sample of permanent residents from the urban area of São Paulo, the most populous city in Brazil and Latin America. Participants were interviewed in their homes by trained researchers using structured questionnaires to obtain comprehensive information on demographic, socioeconomic, lifestyle, and other characteristics. Participants were categorized into three age groups: adolescents (12 to 19), adults (20 to 59), and older adults (60 and older).

A subsample of 901 participants was randomly selected for venous blood sample collection to assess biochemical markers, nutritional status, and genetic biomarkers, in accordance with established health protocols. Blood was collected by trained nurses during visits to the participants’ households, who also conducted anthropometric assessments (weight, height, waist circumference) and blood pressure measurements. Details of data collection and processing have been previously described elsewhere (27).

This study was conducted in accordance with the Declaration of Helsinki and approved by the Ethics Committee on Research of the School of Public Health of the University of São Paulo (#30848914.7.0000.5421).

### 2.2 Genomic data assessment

Genomic DNA was extracted from peripheral blood and stored in buffer containing EDTA at −80 °C. Samples were processed using an automated extraction protocol on the QIAsymphony SP BioRobot with the QIAsymphony DNA Midi Kit 96 (Qiagen, Hilden, Germany), following the manufacturer’s instructions. DNA purification and quality control steps were performed. The Axiom 2.0 Precision Medicine Research Array was used to genotype 846 samples at the Thermo Fisher Scientific laboratory (Affymetrix Inc., Santa Clara, CA). After quality control and exclusion of missing data (5 individuals were excluded), using GRCh37 as the reference genome, 873,177 markers were available for 841 individuals, who may have some level of relatedness, given that they were distributed across 629 households. Detailed protocols on genomic data assessment and quality control can be found elsewhere (25).

### 2.3 Genetic relatedness

Given that some of the 841 individuals shared the same household, suggesting they might be relatives, we calculated a Genetic Relationship Matrix (GRM) to quantify genetic similarity between individuals based on their genotypes, which was later used to exclude closely related participants.

Independence among individuals is an essential assumption for both Hardy-Weinberg equilibrium (HWE) and linkage disequilibrium (LD) analyses, which does not hold for all 841 individuals. First, individuals from the same household who were not spouses were first excluded from the dataset, resulting in a total of 707 individuals. Filters were then applied to this subset, including a minimum allele frequency (MAF) threshold of 0.05, Hardy-Weinberg equilibrium (p-value < 10^-6^), and linkage disequilibrium (LD) pruning (r^2^ > 0.5), to filter SNPs for the estimation of the GRM. Additionally, participants and SNPs with more than 5% missing data were excluded from the analysis. The remaining SNPs selected were then used for all 841 individuals, and the GRM was calculated using the software PLINK 2.0.

### 2.4 Quality control for association analysis and estimation of principal components of ancestry

To perform quality control on the genetic dataset for association studies, one individual from each pair in the pairwise GRM with a genomic relationship coefficient greater than 0.125 was excluded prior to the association analyses. Since this step removed only a relatively small fraction of the sample (121 of 841 individuals), the final analytic dataset excluded all pairs of second-degree or closer relatives and comprised 720 individuals. Quality control filters were then applied to this subset, including a minimum allele frequency (MAF) threshold of 0.05, Hardy-Weinberg equilibrium (p-value < 10^-6^), and linkage disequilibrium (LD) pruning (r^2^>0.5). Once more, individuals and SNPs with more than 5% missing data were excluded from the analysis. As a result of this process, a total of 628,839 SNPs were removed, leaving a final dataset of 244,338 SNPs.

In addition, using 238,816 markers from the array that were also present in common with the 1000 Genomes Project phase 3 (1 KGP) after quality control pruning, principal components of ancestry (PC) were estimated with the SNPRelate package in R software v4.1.0 and PLINK 2.0, as previously described (25). Hence, information on 244,338 SNPs and PCs for 720 independent individuals was available for genetic analysis.

**Figure 1:**
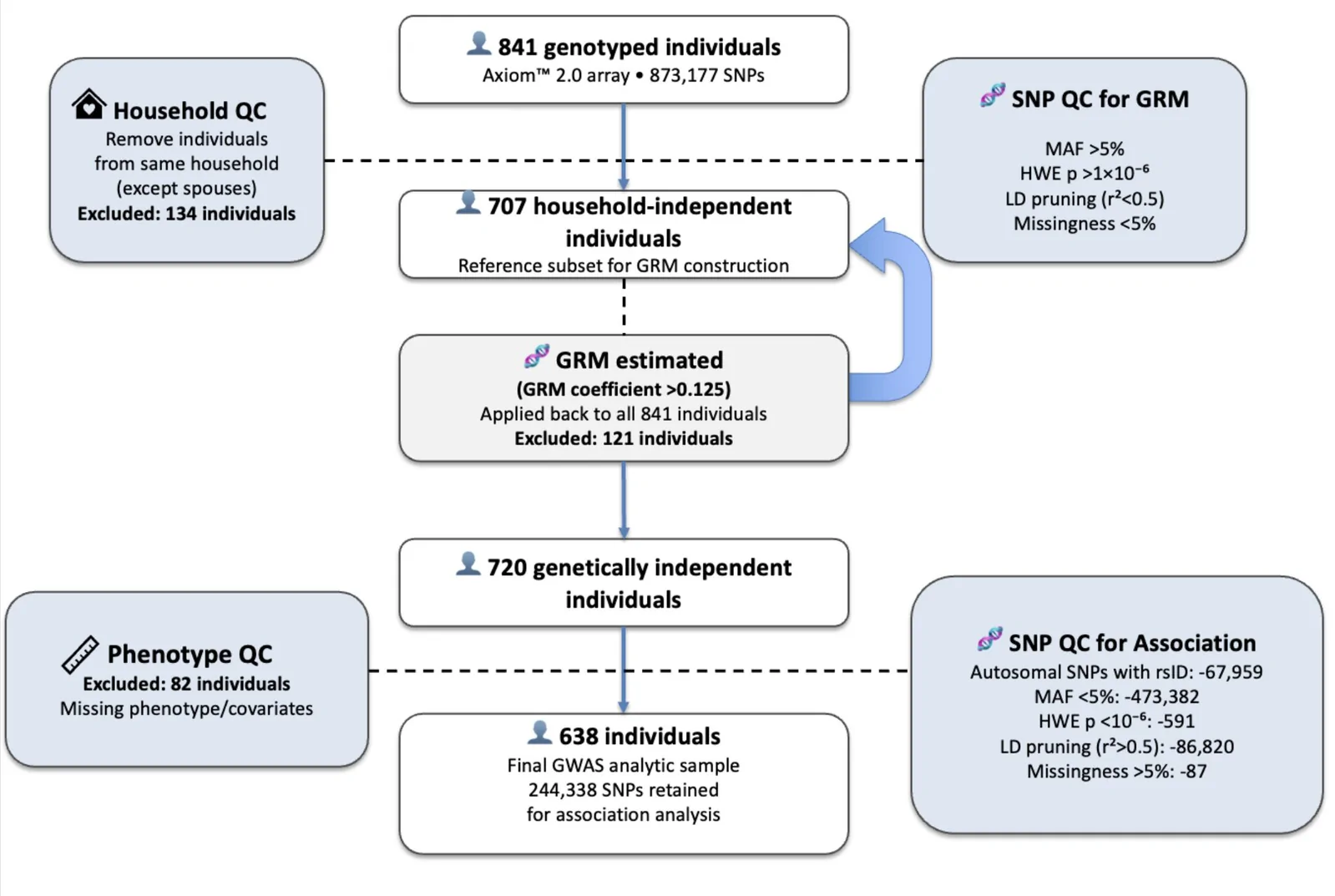
Individual- and SNP-level quality-control workflow for GWAS. QC, quality control; GRM, genetic relationship matrix; GWAS, genome-wide association study; SNP, single nucleotide polymorphism; MAF, minor allele frequency; HWE, Hardy-Weinberg equilibrium; LD, linkage disequilibrium. rsID, reference SNP cluster identification.

The workflow comprising these quality control procedures along with genetic relatedness estimation for GWAS analysis are summarized in **Figure 1**.

### 2.5 Phenotypic variables

The variables of interest in this investigation encompassed a comprehensive set of phenotypes:

- Sociodemographic: Sex, age, age^2^ (age squared), and age group (Adolescents: 12–19 years-old; Adults: 20 to 59 years-old; Older adults: ≥60 years-old). Ethnicity, which corresponds to self-reported skin color/race (based on five standard ethnoracial self-classification groups in Brazil, Black (“Preta”), Indigenous (“Indígena”), Mixed (“Parda”), White (“Branca”), or Yellow (“Amarela”) (28, 29). Ethnicity was ultimately categorized into “White” and “Not white”.
- Adiposity: Body mass index (BMI, kg/m^2^) was used to categorize participants into the following groups: without excess body weight, with excess body weight (adolescents: >+1SD BMI-for-age, adults: 25≤BMI<30 kg/m²; older adults: 28≤BMI<30 kg/m²), or obesity (adolescents: >+2SD BMI-for-age, adults and older adults: BMI≥30 kg/m²) (30–32). If participants had excess body weight or were obese, they were categorized as “Having Overweight (Yes)” vs “Not Having Overweight (No)”.
- Lifestyle: Alcohol use (Yes or No) and Smoking status (Smoked at least once and Never Smoked).
- Inflammation: Interleukin (IL)-1β, IL-6, IL-10, TNF-α, IL-10/TNF-α ratio, CRP, MCP-1 and adiponectin.

To measure inflammatory biomarkers, blood samples were collected after 12 to 14 h of fasting, in EDTA (Ethylenediamine tetra-acetic acid)-containing tubes and sent to the Laboratory of Nutritional Genomics and Inflammation at the Public Health School of the University of São Paulo. Samples were stored at -70 °C for further analysis. Interleukin (IL)-1β, IL-6, IL-10, TNF-α, MCP-1 and adiponectin were measured via a multiplex immunoassay (Milliplex, Merk Millipore, Darmstadt, Germany). The plasma circulating C-reactive protein (CRP) was measured by kinetic turbidimetry using the IMMAGE^®^ immunochemistry system kit (Beckman Coulter Inc., Brea, CA, USA). Further details of procedures can be found in Fisberg et al. (2018).

After data processing and exclusion of observations with missing covariate values, 638 individuals had information available for analysis.

### 2.6 Descriptive analysis

Quantitative variables were described using medians and interquartile ranges, whereas categorical variables were expressed as percentages. Normality of the inflammatory markers was evaluated using histograms, and the Shapiro-Wilk test. The inflammatory biomarkers with non-normal distributions were transformed using the Rank-based inverse normal transformation (INT) with the RankNorm function from the RNOmni package in R for GWAS.

Correlation analysis was performed for inflammatory biomarkers with the Spearman correlation test, with variables ordered using the hclust function in R. Correlation plots were built using the R package ggplot2, and correlation coefficients (absolute values) within the ranges 0–0.39, 0.40–0.69, and 0.70–1 were considered low, moderate, and high, respectively.

### 2.7 GWAS analysis

To evaluate the effect of each of the genetic markers (SNPs), we used the polygenic model of additive effects:


$$y_{i}=\mu +\beta^{\prime }\times X_{i}+\;\beta_{SNP}\times \;X_{SNPi}+\;\epsilon_{i}$$

where y_i_, response variable of the i^th^ individual; µ, overall mean; β’, transposed vector of covariate effects; X_i_, vector of covariates for the i^th^ individual; X_SNPi_, the genotype information for the i^th^ individual; β_SNP,_ SNP effect and ϵ_i_, residual term associated with the i^th^ individual. We assume that i ~ N(0, σ^2ε^), meaning that errors are independently and normally distributed with mean zero and constant variance.

All models were adjusted for the base covariates age, age^2^, sex, overweight (Yes or No) and the main two principal components of ancestry (PC1 and PC2) were included as covariates to control for population stratification. This choice was based on a companion population-genetics analysis of this same cohort, which showed that PC1 and PC2 jointly explained 84.8% of the genetic variance in a combined PCA with 1000 Genomes reference panels, and 35.8% of the variance within the ISA-Nutrition sample alone, reflecting the predominantly two-way (European–African) admixture pattern of this population, with a comparatively minor Native American contribution and negligible East Asian ancestry (25). In order to confirm appropriate control of population structure, we calculated the λGC (genomic control) using R. Model diagnostics (i.e., whether the assumptions of normality and homoscedasticity of residuals are satisfied) were performed with the Shapiro-Wilk and Breusch-Pagan tests, respectively. The quality of the GWAS analyses was further assessed via QQ-plots, and Manhattan plots were generated to display significant SNPs using the qqman R package. Due to our limited sample size, we adopted a suggestive genome-wide significance level of 10^-5^, also depicting the line for the conventional 5 x 10–^8^ genome-wide significance level in the Manhattan plots. Effect estimates were also displayed in a Forest plot built in R.

Additionally, we performed a *post hoc* power analysis using the genpwr R package. For each reported SNP association, we estimated (i) the statistical power corresponding to the observed standardized allelic effect size and (ii) the minimum detectable standardized allelic effect required to achieve 80% power at the suggestive genome-wide significance threshold (α=10^-5^), considering the observed minor allele frequency (MAF) and the effective sample size (N = 638).

All statistical analyses were performed in RStudio v2025.09.1 + 401, annotated information of SNPs was retrieved from the NCBI dbSNP and additional information on the putative biological roles and implications of loci and its respective genome regions were searched in Pubmed and in the GWAS Catalog. Furthermore, we performed functional annotation of missense variants using the Combined Annotation Dependent Depletion (CADD), particularly the CADD model GRCh37-v1.7.

## 3 Results

### 3.1 Descriptive statistics

Summary statistics are presented in **Table 1**. Participants were predominantly male (53%) and had a median age of 49. Most belonged to the older adult group (38%) and were not overweight (55%). The most striking absolute differences were observed for lifestyle characteristics. For instance, 71% reported they never smoked, and 71% did not consume alcohol.

**Table 1:** Sociodemographic and cardiometabolic features of individuals in the 2015 ISA-Nutrition study.

Variable	N = 638
Sex	
Male	336 (53%)
Female	302 (47%)
Age	49 (18, 65)
Age ²	2,401 (324; 4,225)
Age group	
Adolescent	192 (30%)
Adult	205 (32%)
Older adult	241 (38%)
Overweight	
No	349 (55%)
Yes	289 (45%)
Smoking	
Never Smoked	452 (71%)
Smoked at least once	186 (29%)
Alcohol use	
No	491 (77%)
Yes	147 (23%)
Ethnicity	
White	327 (51%)
Not White	311 (49%)
CRP (pg/mL)	293,996 (99,565; 756,337)
Adiponectin (pg/mL)	20,603,648 (10,693,241; 40,393,544)
IL-6 (pg/mL)	1 (1; 3)
IL-1β (pg/mL)	1.16 (0.89; 1.50)
IL-10 (pg/mL)	4.3 (3.0; 6.6)
TNF-α (pg/mL)	11.2 (8.4; 14.4)
MCP-1 (pg/mL)	281 (217; 349)
IL-10/TNF-α	0.40 (0.28; 0.66)
PC1	-0.003 (-0.010; 0.003)
PC2	-0.013 (-0.018; -0.008)

*Median (IQR, interquartile range); N (%) (absolute frequency and proportion); PC, principal component of ancestry; MCP1, monocyte chemoattractant protein; CRP, C-reactive protein; TNF-α, tumor necrosis factor α; IL , interleukin.

**Figure 2:**
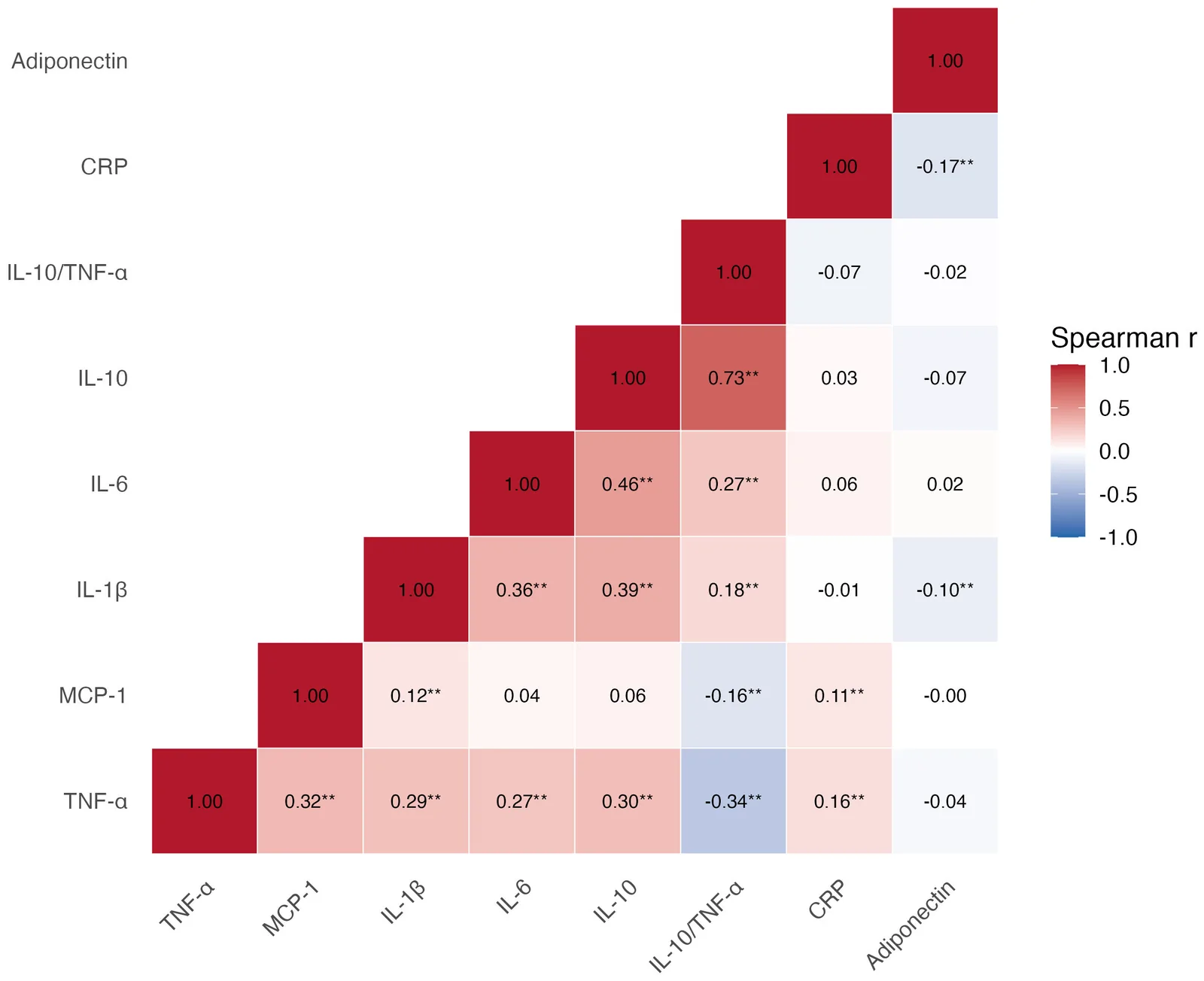
Correlation heatmap of inflammatory biomarkers for 638 participants of the 2015 ISA-Nutrition study. ** corresponds to p < 0.01. CRP, C-reactive protein; TNF, tumor necrosis factor; IL, interleukin; MCP, monocyte chemoattractant protein. N = 638.

Regarding pairwise correlations, except for those between IL-6 and IL-10 (r, 0.46) and between IL-10 and the IL-10/TNF ratio (r, 0.73), all others were low (**Figure 2**).

### 3.2 GWAS

**Figure 3:**
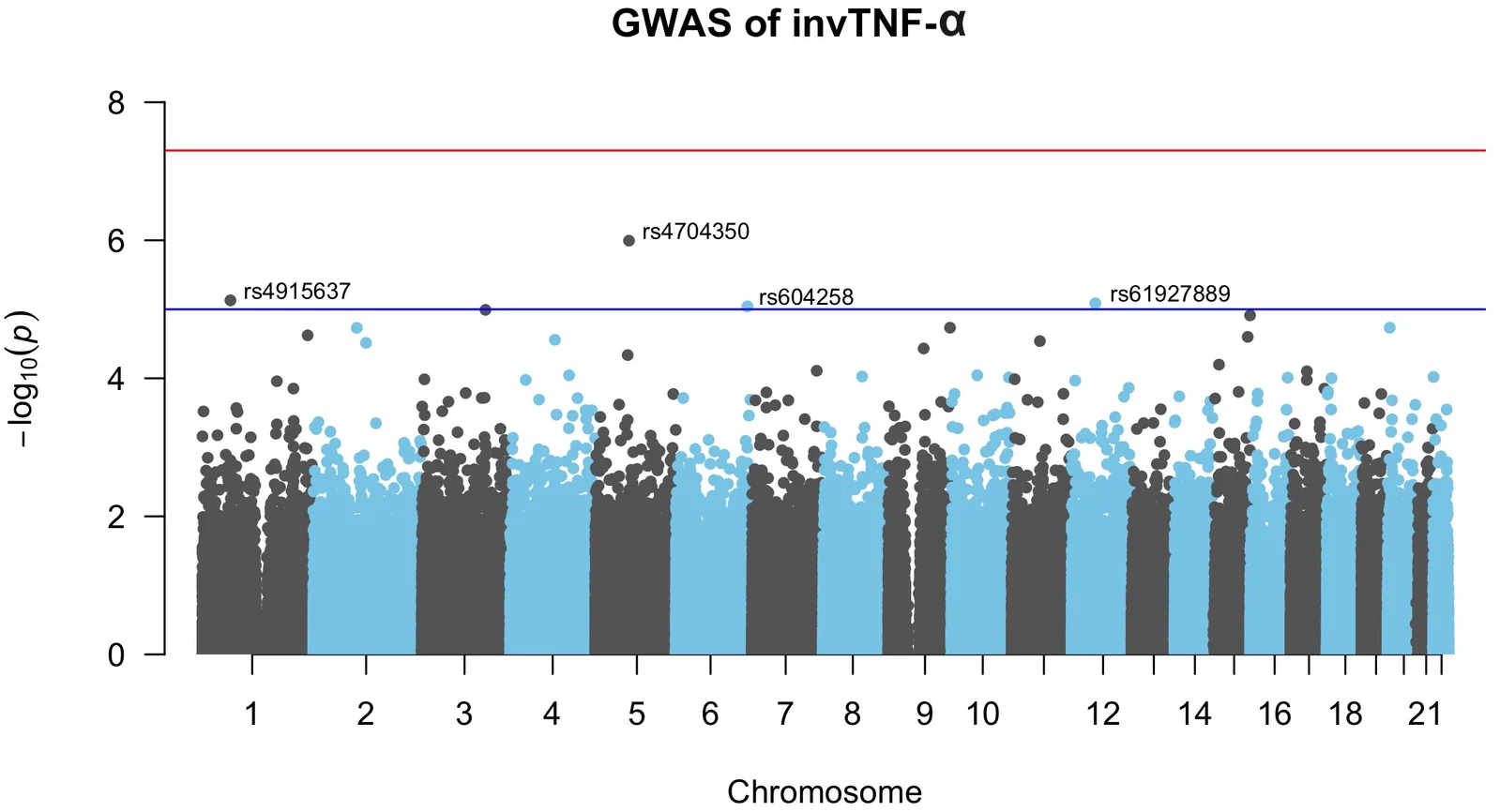
Manhattan plot of significant associations with inverse-normal-transformed TNF-α. The horizontal line corresponds to the suggestive genome-wide significance level of 10^-5^. TNF, tumor necrosis factor. N = 638.

**Figure 4:**
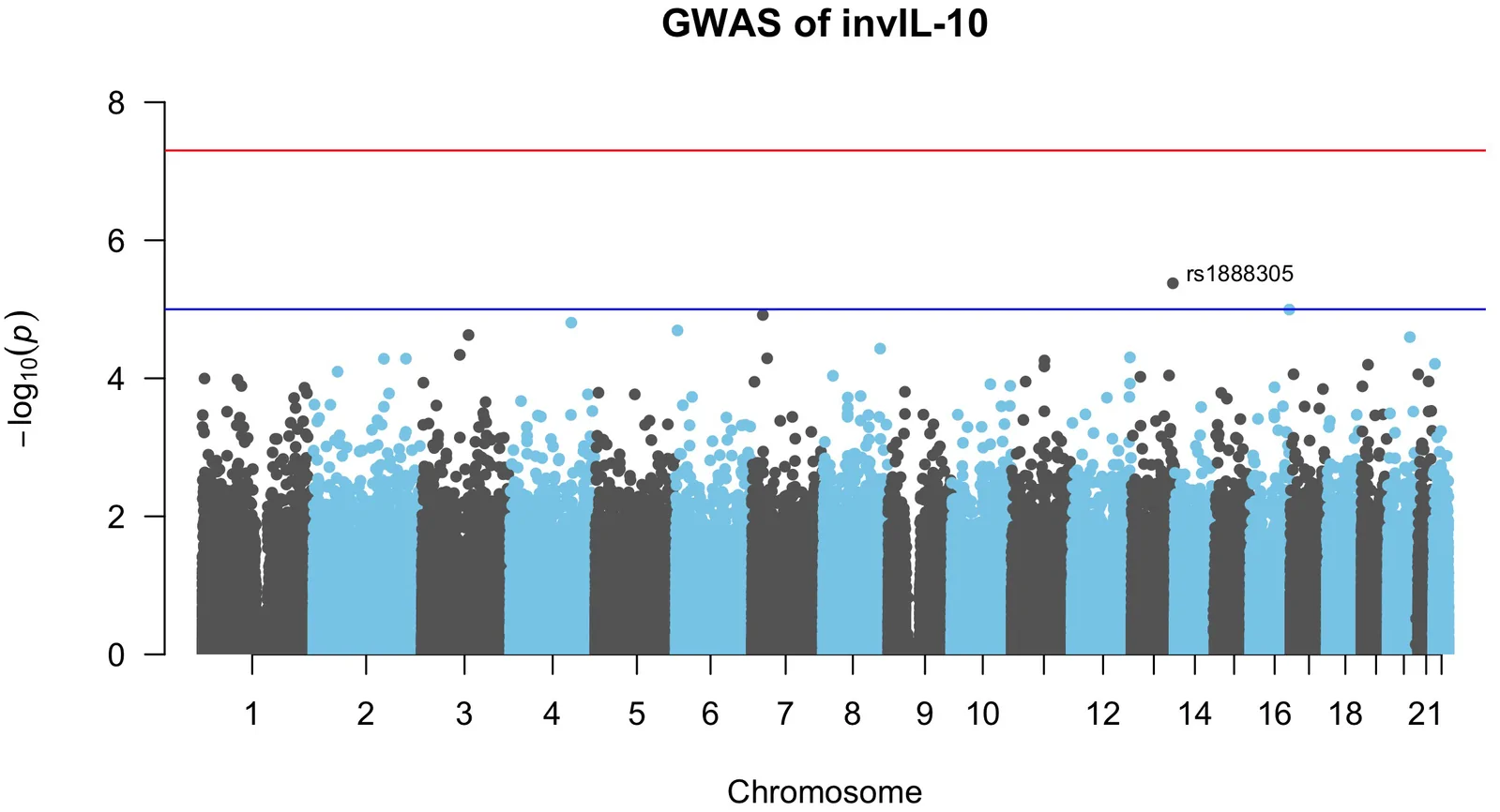
Manhattan plot of significant associations with inverse-normal-transformed IL-10. The horizontal line corresponds to the suggestive genome-wide significance level of 10^-5^. IL, interleukin. N = 638.

**Figure 5:**
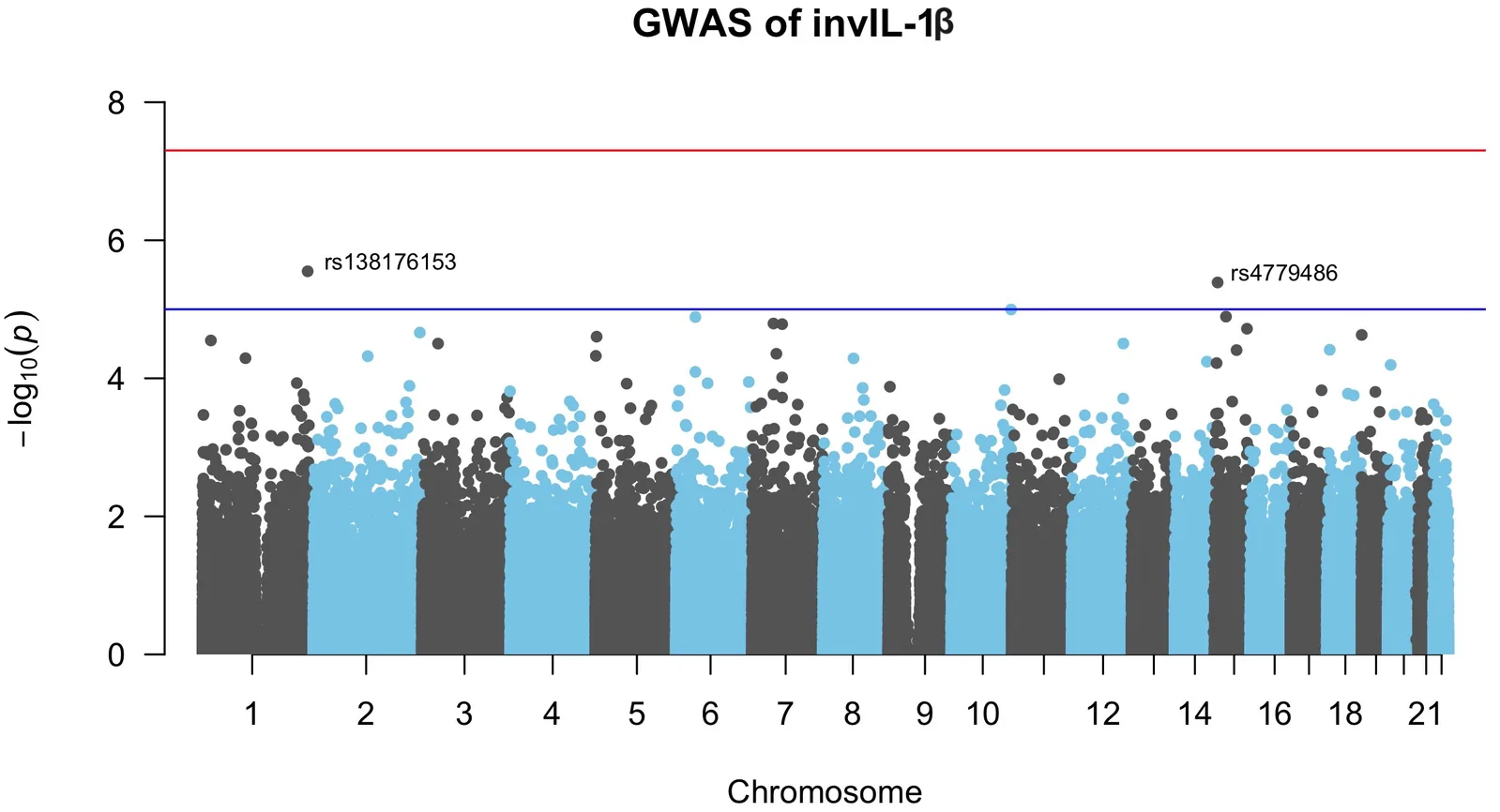
Manhattan plot of significant associations with inverse-normal-transformed IL-1β. The horizontal line corresponds to the suggestive genome-wide significance level of 10^-5^. IL, interleukin. N = 638.

**Figure 6:**
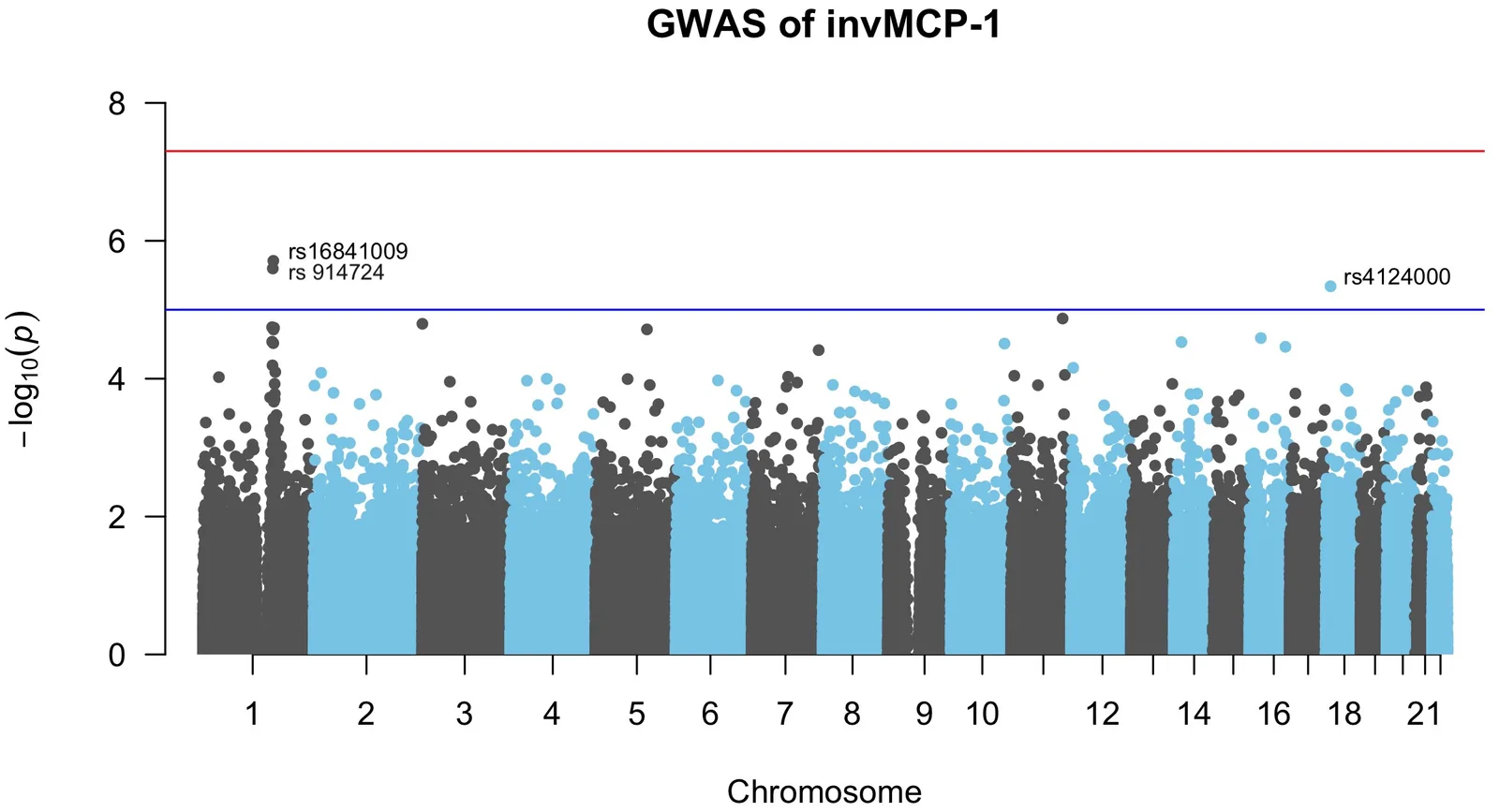
Manhattan plot of significant associations with inverse-normal-transformed MCP-1. The horizontal line corresponds to the suggestive genome-wide significance level of 10^-5^. MCP, monocyte chemoattractant protein. N = 638.

**Figure 7:**
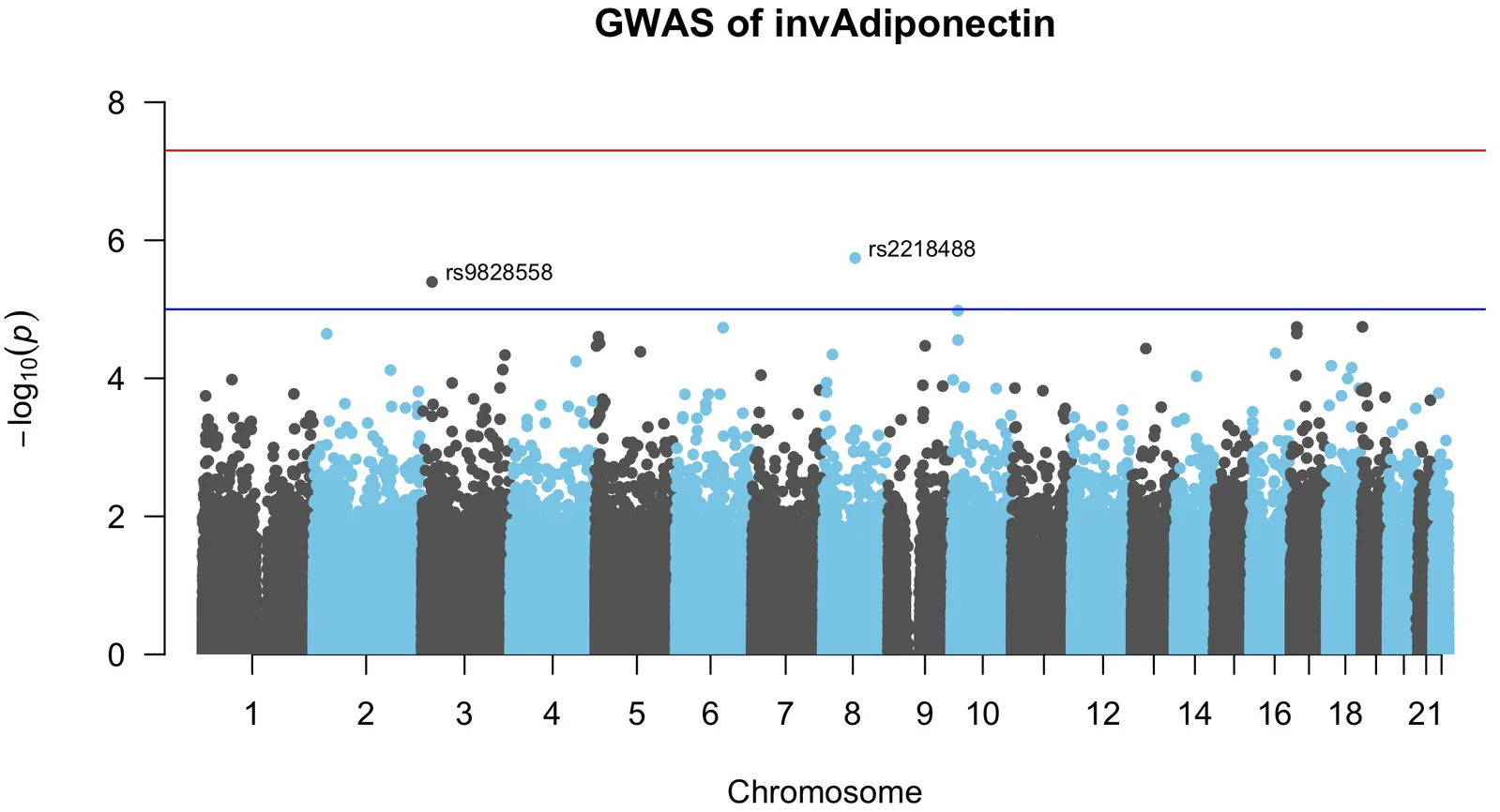
Manhattan plot of significant associations with inverse-normal-transformed adiponectin. The horizontal line corresponds to the suggestive genome-wide significance level of 10^-5^. N = 638.

For the three biomarkers IL-6, IL-10 and CRP, the assumptions of the linear model were not met and the quality control assessment via QQ-plots showed evidence of systematic errors in the p-values estimation. Hence, only TNF-α, IL-10, IL-1β, MCP-1, and adiponectin (**Supplementary Material 1**) showed no evidence of neither systematic error nor any deviation from the assumptions of normality of residuals and homoscedasticity and could be further analyzed. The genome-wide analysis of these 5 inflammatory biomarkers showed 12 SNPs significantly associated (**Figure 3**–**7**) under the suggestive genome-wide significance level, whose detailed information is provided in **Table 2**.

**Table 2:** Significant associations of SNPs with inflammatory biomarkers.

SNP	CHR	EA	β coefficient	SE	CI95 Lower	CI95 Upper	p-value	MAF	Phenotype	Gene: consequence
rs4915637	1	G	-0.248	0.055	-0.356	-0.140	7.42E-06	0.35	invTNF- α	*LINC00466*: Intron Variant
rs4704350	5	A	0.430	0.087	0.259	0.601	1.01E-06	0.12	invTNF- α	*IQGAP2*: Intron Variant
rs604258	6	C	0.308	0.069	0.173	0.443	9.09E-06	0.20	invTNF- α	*LOC105378088*: Non Coding Transcript Variant
rs61927889	12	G	0.254	0.056	0.143	0.364	8.20E-06	0.38	invTNF- α	*KRT8*: Intron Variant
rs1888305	13	T	0.276	0.060	0.160	0.393	4.19E-06	0.27	invIL-10	None
rs138176153	1	TTTAT	-0.569	0.120	-0.805	-0.332	2.82E-06	0.06	invIL-1β	None
rs4779486	15	G	-0.280	0.060	-0.398	-0.162	4.08E-06	0.38	invIL-1β	*APBA2*: Intron Variant
rs914724	1	G	-0.290	0.061	-0.410	-0.170	2.52E-06	0.30	invMCP-1	*FCRL5*: Intron Variant
rs16841009	1	C	-0.455	0.095	-0.641	-0.269	1.95E-06	0.09	invMCP-1	*OR6K6*: Missense Variant
rs4124000	18	G	0.258	0.056	0.148	0.367	4.57E-06	0.35	invMCP-1	*PIEZO2*: Intron Variant
rs9828558	3	G	0.232	0.050	0.134	0.330	4.02E-06	0.362	invAdiponectin	None
rs2218488	8	C	0.332	0.069	0.197	0.468	1.81E-06	0.147	invAdiponectin	*EYA1*: Intron Variant

SNP, single nucleotide polymorphism; CHR, chromosome; MAF, minor allele frequency; SE, Standard error; CI95, 95% confidence interval boundaries. The Gene: consequence information was retrieved from the dbSNP.

**Figure 8:**
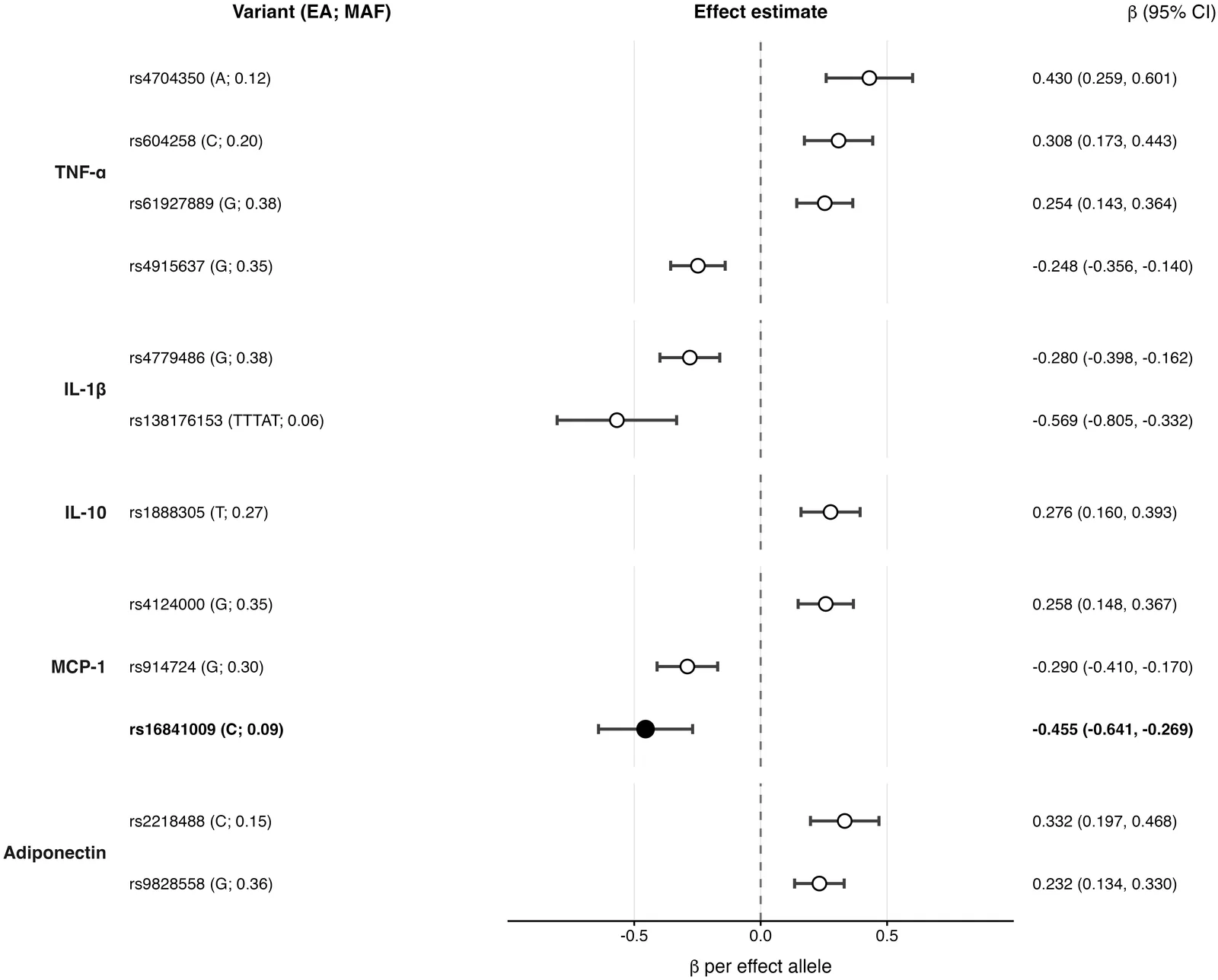
Effect estimates for suggestive GWAS associations with inflammatory biomarkers. Points represent per-effect-allele regression coefficients; Horizontal lines represent 95% confidence intervals; The filled point highlights the missense variant rs16841009; EA, effect allele; MAF, minor allele frequency; TNF, tumor necrosis factor; IL, interleukin; MCP, monocyte chemoattractant protein; Outcomes were rank-based inverse-normal transformed.

TNF-α exhibited the highest number of associations (four), as opposed to IL-10 (one), while IL-1β had two associated SNPs, adiponectin had two and MCP-1 had three. Most loci corresponded to intron variants, with MAF ranging from 0.06 to 0.38. Notably, one SNP associated with MCP-1 corresponded to a missense variant, with a negative beta coefficient. The absolute effect of this variant was the second highest (0.455), while its MAF was the second lowest (0.09) (**Table 2**). Across phenotypes, there were positive beta coefficients for seven SNPs and negative coefficients for the remaining five (**Table 2**; **Figure 8**). The reported associations showed observed power below the conventional 80% threshold and the test for the missense variant achieved 60.8% power (**Supplementary Material 1**).

Moreover, functional annotation with CADD of the missense variant associated with MCP-1 indicated a Phred-scaled score of 24.8.

## 4 Discussion

To provide insights into the genetic architecture of inflammatory biomarkers, we conducted several exploratory GWAS, which indicated multiple suggestive associations of SNPs with these phenotypes in a sample of 638 individuals from a highly admixed and underrepresented population in Brazil. Most significantly associated loci were mapped to intronic regions, including of a non-coding RNA, and some associations had no reported gene consequence. In addition, a noteworthy result was a missense variant that showed a negative association with MCP-1 plasma concentrations.

The MCP1-associated missense variant rs16841009 is a T>C substitution that results in the amino acid change Cys > Arg, position 173, and lies within the gene OR6K6, which encodes the olfactory receptor (OR) family 6 subfamily K member 6, also known as OR1-21. Notably, ORs are the largest family of G-Protein-coupled receptors and, beyond their canonical role in olfaction, there is growing evidence of ORs extra nasal functions due to their ectopic expression in extra-olfactory tissues and cells, including macrophages. These receptors can interact with dietary patterns (e.g., high-fat diet, fruit and tea intake), gut microbiota-derived metabolites (e.g., acetate and propionate), and molecules involved in oxidative stress (e.g., octanal), which may have implications for several diseases such as cancer, obesity, and atherosclerosis (33–36).

Recently, Ehlinger J.V. et al. (2023) conducted an epigenome-wide association study of blood leucocytes from children with Attention-deficit/hyperactivity disorder (ADHD). They found significant associations between cognitive outcomes and differentially DNA methylation of three loci, including OR6K6, as well as enrichment of pathways related to neurotransmission, immune response and inflammation (37). Yet, to the best of our knowledge, the present study is the first GWAS to report an association between rs16841009 and circulating MCP-1. Neither this variant nor OR6K6 has been previously implicated in inflammatory traits according to the GWAS Catalog or dbSNP. Although direct evidence linking OR6K6 to MCP-1 regulation is lacking, prior studies have demonstrated that other olfactory receptors can enhance LPS- and IFN-γ–induced MCP-1 expression in macrophages (38). Moreover, OR family members have been implicated in modulating inflammatory and metabolic conditions, with mechanisms that include inflammasome (NLRP3) activation, cytokine dysregulation, and M2 macrophage polarization (33–35).

To assess further evidence on the potential biological relevance of the p.Cys173Arg substitution in OR6K6, we performed functional annotation with CADD. The assigned Phred score of 24.8 for this variant indicates that it ranks among the top 1% of predicted functionally impactful substitutions in the human genome according to the CADD framework (39). Taken together with the aforementioned literature evidence, these observations support the hypothesis of an immunomodulatory role for OR6K6; however, additional functional studies are required to determine whether rs16841009 directly influences MCP-1 expression or only reflects linkage disequilibrium with regulatory variants in the locus.

Regarding intron variants, special consideration should be given to the one in the LINC00466 gene, which codes for the long intergenic non-coding RNA (ncRNA) 466. It has been extensively demonstrated that ncRNAs play important regulatory roles in T2D and associated metabolic conditions, cardiovascular diseases (CVD), and several cancers (40–42). For instance, it was recently shown that LINC00466 promotes the progression of breast cancer via the miR-4731-5p/EPHA2 pathway (43). Interestingly, miR-4731-5p has been shown to participate in a competing endogenous RNA (ceRNA) network that is involved in pulpitis, an inflammatory condition (44). Similarly to the findings in the mentioned breast cancer study, other studies reported that this ncRNA 466 affects the prognosis and progression of several cancers by miRNA modulation (45, 46). Nonetheless, no previous study has shown a direct role of LINC00466 in inflammatory traits in either high or low-grade inflammation contexts.

Herein, the suggestive genome-wide association between rs4915637 in LINC00466 and circulating TNF-α provides novel evidence for potential regulatory roles of this lncRNA in the context of inflammation and immune modulation. However, the results should be interpreted with caution, as further investigations are warranted to assess the consistency of this association and the molecular mechanisms underlying the relationship between TNF-α and lncRNA 466.

This investigation has numerous strengths. First, it adopted strict methodological rigor regarding data collection and analysis, including robust assessment of fundamental statistical assumptions and quality control in GWAS. Second, the study evaluated a Brazilian cohort that is representative at the population level in the country’s largest city and, importantly, considered the miscegenated nature of the population through adjustment by principal components of ancestry. The Brazilian population remains underrepresented in genetic research despite being one of the most admixed population in the world. In fact, recent research using sequencing data from Brazilians across all their five geographical regions identified more than 8 million previously unknown variants, with a positive correlation with genetic ancestry components (24). Taken together, these aspects strengthen the study’s contribution to the understanding of genes and other genome regions involved in health and disease, with particular emphasis on inflammatory cytokines, which may have important biological implications.

Nonetheless, several limitations should be acknowledged. First, this is an exploratory study with an observational, cross-sectional design, which does not allow for causal inference, limiting the clinical significance of the results. Also, external replication was not available. Moreover, although additional inferential and PCA analysis of inflammatory biomarkers showed little difference across age groups (Results not shown), there might still be potential heterogeneity due to the combination of adolescents and older individuals in a single GWAS. In addition, across-studies differences in the set of evaluated SNPs due to lack of imputation or to the genotyping technique (either array or sequencing technologies), adjustment covariates included in the GWAS analysis, population structure due to distinct genetic admixture, among others, may hinder the comparison of our findings with those of other studies. Notably, we used BMI as a proxy of adiposity instead of employing measures of abdominal adiposity such as waist circumference. Although these measures may be more closely related to inflammation rather than BMI alone, BMI is still the most used adiposity variable for adjustment in GWAS and its use in the present study can facilitate integration with other data sets in further studies. Yet, further GWAS investigations testing the influence of other adiposity measures in inflammatory biomarkers should be encouraged.

Furthermore, it should be noted that the presented loci were identified under a suggestive genome-wide threshold, which means that their significance should be considered with additional caution. Notably, this study had a limited sample size, which led to reduced power to detect true positive associations between the evaluated SNPs and inflammatory phenotypes, as shown by the *post hoc* power analysis. Yet, one of the largest observed powers among the significant SNPs corresponded to the missense variant rs16841009. Interestingly, rs16841009 also exhibited one of the largest estimated effect sizes despite having one of the lowest minor allele frequencies among the reported loci. Although these estimates should be interpreted cautiously given the limited sample size, this observation suggests that relatively uncommon variants with moderate-to-large effects may contribute to inter-individual variation in circulating MCP-1 levels.

However, further investigations with larger sample sizes, other modeling approaches such as mixed models, external replication, the assessment of potential sources of heterogeneity, together with other functional analyses, are still necessary to provide clearer and more precise details of the molecular mechanisms by which SNPs, especially the missense variant in *OR6K6*, may impact inflammatory cytokines and cardiometabolic conditions.

## 5 Conclusion

This study provided novel suggestive evidence of the common genetic variants associated with variation in inflammatory biomarkers in an underrepresented and highly admixed Brazilian population. Alongside the ncRNA variant rs4915637 in LINC00466, the missense variant rs16841009 in the olfactory receptor gene OR6K6 was found to be associated with MCP-1, which, considering robust literature reports on the relationship between ectopically expressed olfactory receptor and inflammatory functions, suggests this novel association might play a biological role in inflammatory processes with potential clinical relevance for cardiometabolic diseases.

Nonetheless, the findings should be considered with caution and further investigations to replicate, confirm these findings and elucidate the roles of olfactory receptors and ncRNAs in immune and inflammatory mechanisms are encouraged and may be achieved through fine-mapping, functional annotation, and as well as candidate-gene experiments.

## Data Availability

The genetic datasets analyzed during the current study are available in the European Genome-phenome Archive (EGA) repository (https://ega-archive.org/ (accessed on 30 March 2026)), (study ID EGAS00001007818) under request. Requests to access these datasets should be directed to https://ega-archive.org/, study ID EGAS00001007818 or, alternatively, to Prof Dr. RF (regina.fisberg@gmail.com).
