# Supplementary material for: Suggestive genome-wide associations with inflammatory biomarkers in an admixed population, including a missense variant in the OR6K6 olfactory receptor gene associated with MCP-1
Source: Front Immunol. 2026 Aug 25;17:1876244. doi: 10.3389/fimmu.2026.1876244 (PMC13549854; doi:10.3389/fimmu.2026.1876244)
Supplement: Supplementary file 1 [file SupplementaryFile1.docx]

Supplementary Material

# 1 Supplementary Tables and Figures

## 1.1 Supplementary Tables

Table S1. Model diagnostics of base models of inflammatory biomarkers.

| **Variable** | **R²** | **Adj R²** | **Shapiro-Wilk p-value** | **Breusch-Pagan p-value** | **Assumptions OK** |
| --- | --- | --- | --- | --- | --- |
| invCRP | 0.229 | 0.222 | 0.001 | 0.257 | FALSE |
| invAdiponectin | 0.222 | 0.214 | 0.831 | 0.028 | FALSE* |
| invIL-10 | 0.061 | 0.052 | 0.354 | 0.121 | TRUE |
| invIL-10/TNF-α | 0.040 | 0.031 | 0.696 | 0.035 | FALSE |
| invIL-1β | 0.020 | 0.011 | 0.985 | 0.285 | TRUE |
| invIL-6 | 0.041 | 0.032 | 0.893 | 0.007 | FALSE |
| invMCP-1 | 0.068 | 0.059 | 0.803 | 0.739 | TRUE |
| invTNF-α | 0.085 | 0.077 | 0.931 | 0.190 | TRUE |

* Adiponectin model assumptions were met after the inclusion of SNPs in the GWAS. In all GWAS. assumptions for the models with each SNP were met, unless otherwise specified. CRP = c-reactive protein; MCP = monocyte chemoattractant protein; TNF = tumor necrosis factor; IL = interleukin.

Table S2. Post hoc Power Analysis of Detectable Effect Sizes for suggestive genome-wide significant SNPs associated with inflammatory biomarkers.

| **SNP** | **MAF** | **Observed β** | **Observed abs. β** | **Detectable β 80% Power Alpha 1e5** | **Observed power(%)** | **Difference abs. β and detectable β 80% Power** | **Detected with 80% power** |
| --- | --- | --- | --- | --- | --- | --- | --- |
| rs4915637 | 0.35 | -0.248 | 0.248 | 0.30534 | 43.6 | -0.05734 | FALSE |
| rs4704350 | 0.12 | 0.43 | 0.43 | 0.44817 | 73.4 | -0.01817 | FALSE |
| rs604258 | 0.2 | 0.308 | 0.308 | 0.36411 | 50.7 | -0.05611 | FALSE |
| rs61927889 | 0.38 | 0.254 | 0.254 | 0.30004 | 50.8 | -0.04604 | FALSE |
| rs1888305 | 0.27 | 0.276 | 0.276 | 0.32804 | 49.7 | -0.05204 | FALSE |
| rs138176153 | 0.06 | -0.569 | 0.569 | 0.61324 | 67.5 | -0.04424 | FALSE |
| rs4779486 | 0.38 | -0.28 | 0.28 | 0.30004 | 68.6 | -0.02004 | FALSE |
| rs914724 | 0.3 | -0.29 | 0.29 | 0.31781 | 64.5 | -0.02781 | FALSE |
| rs16841009 | 0.09 | -0.455 | 0.455 | 0.50889 | 60.8 | -0.05389 | FALSE |
| rs4124000 | 0.35 | 0.258 | 0.258 | 0.30534 | 50.5 | -0.04734 | FALSE |
| rs9828558 | 0.36 | 0.232 | 0.232 | 0.30341 | 33.9 | -0.07141 | FALSE |
| rs2218488 | 0.15 | 0.332 | 0.332 | 0.40786 | 43.9 | -0.07586 | FALSE |

SNP – single nucleotide polymorphism; MAF – minor allele frequency; Abs. – Absolute value.

Table S3. Estimation of λGC inflation factor for the GWAS of inflammatory biomarkers.

| **Variable in GWAS** | **λGC inflaction factor** |
| --- | --- |
| invCRP | 1.001 |
| invAdiponectin | 1.011 |
| invIL-10 | 0.989 |
| invIL-10/TNF-α | 0.990 |
| invIL-1β | 1.021 |
| invIL-6 | 1.030 |
| invMCP-1 | 1.015 |
| invTNF-α | 1.001 |

## GWAS = genome-wide association study; Inv = inverse-normal transformed phenotype. CRP = c-reactive protein; MCP = monocyte chemoattractant protein; TNF = tumor necrosis factor; IL = interleukin; GC = genomic control.

## 1.2 Supplementary Figures

**Supplementary Figure 1.** QQ-plots of the p-values from the Manhattan plots of inflammatory biomarkers.
